# Necrotizing sarcoid granulomatosis simulating pulmonary malignancy: A case report

**DOI:** 10.1097/MD.0000000000028208

**Published:** 2021-12-10

**Authors:** Jun Hyeok Kim, Bo Da Nam, Jung Hwa Hwang, Dong Won Kim, Ki-Up Kim, Young Woo Park

**Affiliations:** a Department of Radiology, Soonchunhyang University Seoul Hospital, Seoul, Republic of Korea; b Department of Pathology, Soonchunhyang University Seoul Hospital, Seoul, Republic of Korea; c Department of Allergy and Respiratory Medicine, Soonchunhyang University Seoul Hospital, Seoul, Republic of Korea; d Department of Cardiothoracic Surgery, Soonchunhyang University Seoul Hospital, Seoul, Republic of Korea.

**Keywords:** multiple pulmonary nodule, pulmonary neoplasm, sarcoidosis, vasculitis

## Abstract

**Rationale:**

Necrotizing sarcoid granulomatosis (NSG) has recently been termed “sarcoidosis with NSG pattern” for the disease entity representing nodular sarcoidosis with granulomatous pulmonary angiitis. It is characterized by sarcoid-like granulomas, vasculitis, and a variable degree of necrosis. Its rarity and nonspecific clinical symptoms can easily lead to misdiagnosis or delayed diagnosis.

**Patient concerns:**

We report a 67-year-old female with a biopsy-confirmed sarcoidosis with NSG pattern mimicking pulmonary malignancy on initial chest computed tomography scan.

**Diagnoses:**

Sarcoidosis with NSG pattern.

**Interventions:**

The patient underwent video-assisted thoracoscopic surgery with a lung biopsy. No further treatment was performed after the lung biopsy.

**Outcomes:**

Follow-up imaging studies revealed spontaneous regression of the disease after 2 months.

**Lessons:**

Awareness of this rare benign disease entity and overlapping radiologic manifestations with pulmonary malignancy or other granulomatous diseases can be helpful for making a precise diagnosis with a better differential diagnosis.

## 1 Introduction

Necrotizing sarcoid granulomatosis (NSG) was first described as a rare granulomatous pulmonary disease of unknown etiology in 1973 by Liebow and The.^[1]^ Pathological findings of NSG are characterized by exuberant vascular granulomas with infiltration and occlusion of pulmonary vessels accompanied by large necrosis of lung tissue.^[2–5]^ However, the histology of granulomatous pulmonary angiitis is nonspecific. It can be easily found in various conditions such as infection and foreign body reaction.^[6]^ NSG is no longer a term for a disease entity, but a pathologic finding. It is being replaced by “sarcoidosis with NSG pattern.”^[4,7,8]^ Usual clinical manifestations of sarcoidosis with NSG pattern are nonspecific. On imaging evaluation, sarcoid-like granuloma can be presented as solitary or multiple pulmonary nodules or masses similar to other granulomatous diseases.^[9,10]^ Therefore, it is difficult to differentiate NSG clinically and radiologically from malignancies and other granulomatous diseases. Herein, we report a case of sarcoidosis with NSG pattern mistakenly diagnosed as malignancy on initial evaluation. Differential characteristics of sarcoidosis with NSG pattern in comparison with pulmonary malignancy and other granulomatous diseases are discussed.

## 2 Case presentation

This study was approved by the Institutional Review Board of our institution and the requirement for informed consent was waived (IRB No. SCHUH 2021-10-020).

A 67-year-old female was referred to our pulmonology outpatient clinic due to incidental abnormality on screening evaluation. She was a never-smoker with diabetes mellitus. She had no respiratory or systemic symptoms. Her vital signs were stable with a blood pressure of 121/76 mm Hg, a pulse of 81/min, a respiration rate of 18/min, and a body temperature of 36.5°C. Initial laboratory findings were also unremarkable, which revealed normal ranges of white blood cell count at 6900/μL (normal, 4000–10,000/μL) and C-reactive protein at 0.04 mg/dL (normal, 0.0–0.5 mg/dL).

**Figure 1:**
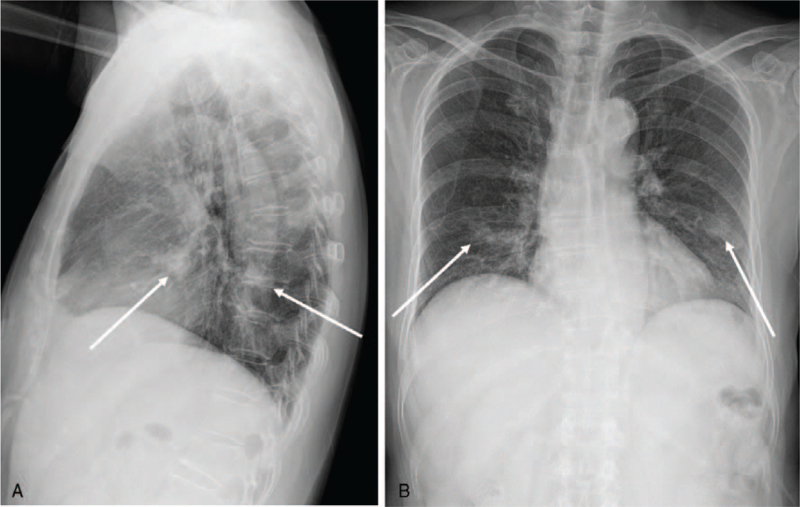
An initial chest radiography for a 67-year-old woman. (A,B) Initial chest radiographs showing lobulated nodular opacities in the right middle lobe and the left lower lobe (arrows).

**Figure 2:**
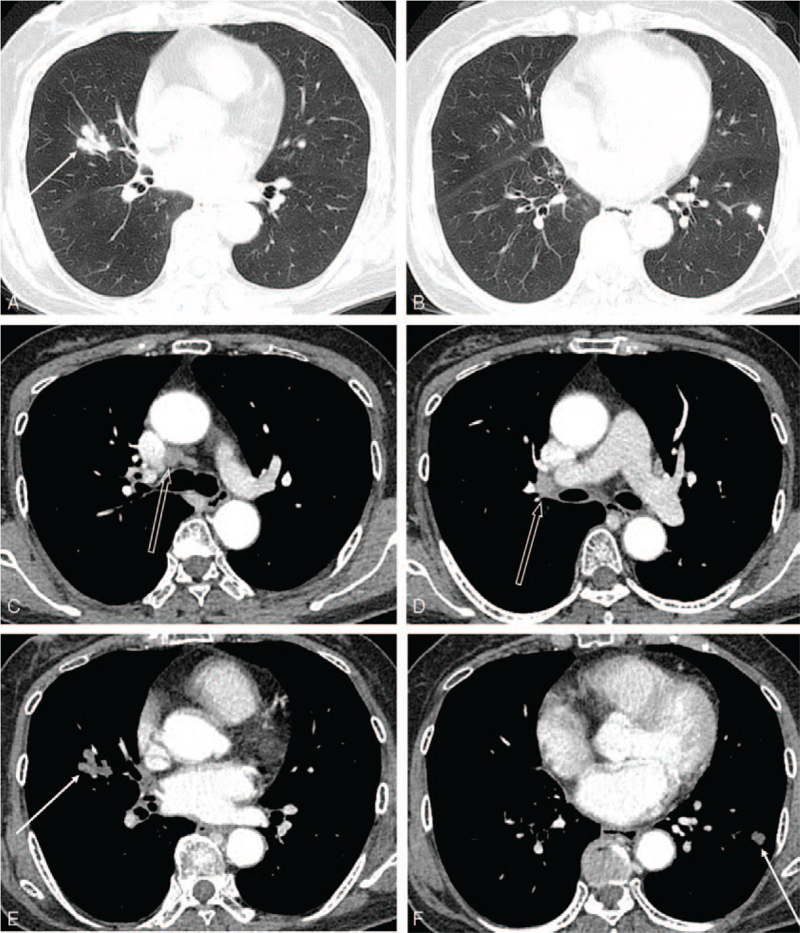
An initial contrast-enhanced chest CT performed at referring center two weeks before visiting our hospital. (A,B) On lung window setting, 2 lobulated nodules are seen in the right middle lobe and the left lower lobe (arrows). (C—F) On mediastinal window setting, there is right lower paratracheal and right hilar lymphadenopathy with homogenous enhancement without necrosis (open arrows in C,D). Contrast enhancement of the nodules in the right middle lobe and the left lower lobe is seen (arrows in E,F). The nodule in the right middle lobe shows peripheral lobulation and air-bronchogram. Another solid nodule in the left lower lobe also shows slight lobulation.

Initial chest radiographs showed 2 less than 2 cm sized lobulated nodular opacities in the right middle lobe and the left lower lobe (Fig. 1), which simulated synchronous multiple primary lung cancers. On review of a contrast-enhanced chest computed tomography (CT) scan performed at the referring hospital 2 weeks ago, about 2 cm sized a lobulated nodule in the right middle lobe and another smaller nodule in the left lower lobe were identified, which showed heterogeneous enhancement with suspicious central low density (Fig. 2). The nodule in the right middle lobe showed air-bronchogram with a slightly dilated bronchus. Slightly enlarged and homogenously enhancing lymph nodes were seen at right lower paratracheal and right hilar areas without necrosis. Our first impression was malignancy such as synchronous multiple primary lung cancers and mucosa-associated lymphoid tissue lymphoma with multiplicities. Indolent infection including fungus or tuberculosis and noninfectious inflammation such as vasculitis, sarcoidosis, and amyloidosis were also included in radiologic differential diagnoses.

**Figure 3:**
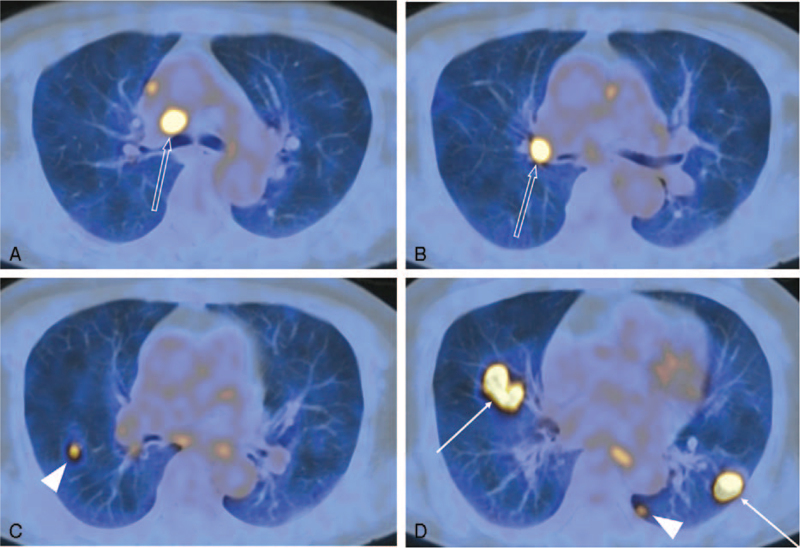
A fluorine 18-fluorodeoxyglucose (FDG) positron emission tomography-computed tomography scan performed at our hospital at 10 days after the initial visit. (A—D) FDG uptakes in the right lower paratracheal and right hilar lymphadenopathy are shown (open arrows in A,B). Avid FDG uptakes are also noted in the nodules in the right middle lobe and the left lower lobe (maximum standardized uptake values: 10.0 and 8.5, respectively) (arrows in D). Other 2 small hypermetabolic nodules are newly revealed in the right middle lobe and the left lower lobe (arrowheads in C,D).

With consideration of malignant tumors, a fluorine 18-fluorodeoxyglucose (FDG) positron emission tomography-CT (PET-CT) scan was performed at 10 days after the initial visit to our hospital. Increased FDG uptakes were noted in the nodules of the right middle lobe and the left lower lobe (maximum standardized uptake values: 10.0 and 8.5, respectively). There were newly seen hypermetabolic nodules in both lower lobes on PET-CT scan (Fig. 3). Right lower paratracheal and right hilar lymphadenopathy also revealed increased FDG uptake. Avid FDG uptakes of the pulmonary nodules could be seen not only in malignancy, but also in active inflammation. Thus, further characterization of the newly appeared lung nodules was necessary.

**Figure 4:**
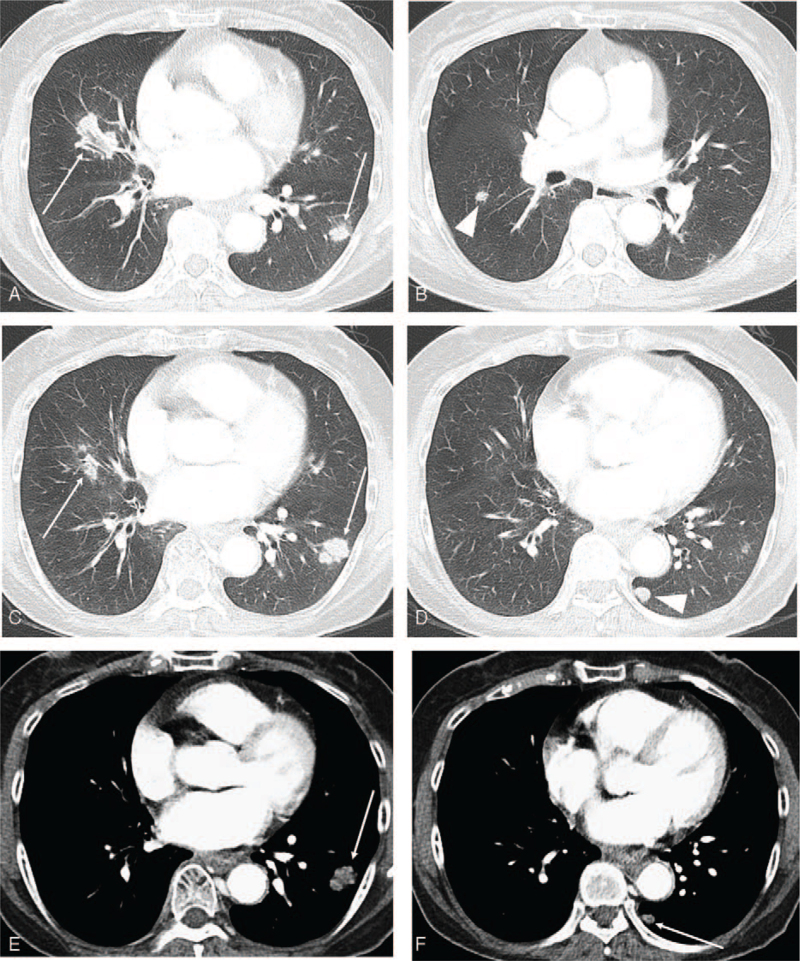
A follow-up contrast-enhanced chest CT performed at our hospital at 1 month after the initial chest CT scanning at the referring center. (A—D) On lung window setting, enlargement of pulmonary nodules (arrows) and newly appeared nodules in the right middle lobe and the left lower lobe are seen (arrowheads). Peribronchovascular distribution of the pulmonary nodules is well demonstrated (arrows). (E,F) On mediastinal window setting, nodules in left lower lobe show areas of low attenuation (arrows) representing necrosis.

A follow-up contrast-enhanced chest CT scan was performed in our institution, which was obtained a month after the initial outside chest CT. Follow-up CT showed enlargement of existing nodules in the right middle lobe and the left lower lobe (from 2 to 2.7 cm and from 1 to 2 cm, respectively) with central low-density necrosis. About 1 cm sized ovoid nodules were newly seen in both lower lobes with a relatively smooth peripheral margin and peribronchovascular distribution of pulmonary nodules was suspected (Fig. 4). Considering radiographic change on chest CT scans during a month of follow-up period, inflammatory diseases were more likely to be the diagnosis than malignancy. Radiologic differential diagnosis included granulomatosis with polyangiitis (GPA) and nodular sarcoidosis. Indolent infection was less likely to be the diagnosis.

**Figure 5:**
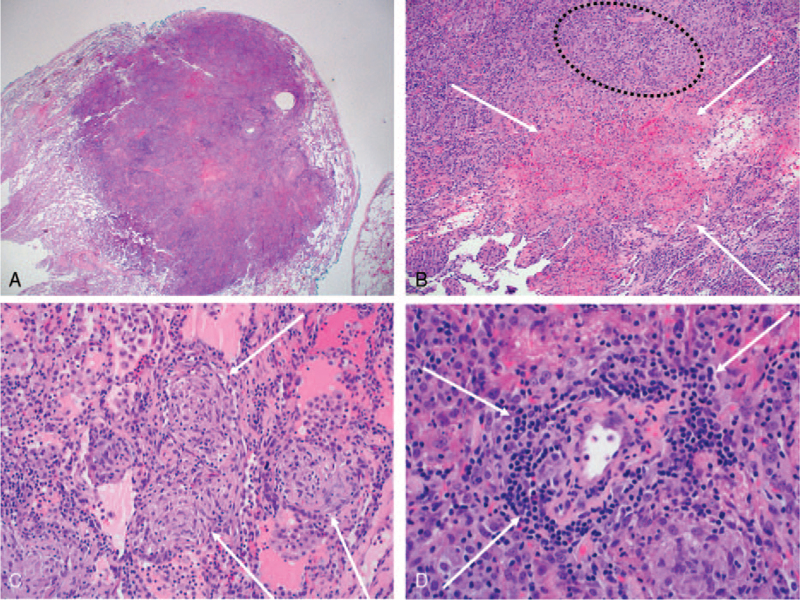
Pathologic findings of a lung specimen obtained from video-assisted thoracoscopic surgery of the left lower lobe. (A) On microscopy, the lung nodule in left lower lobe shows exuberant inflammatory nodules with multifocal necrosis (hematoxylin staining, × 1.25). (B) Large zone of necrosis (arrows) and multiple granulomas (dotted circle) are seen (hematoxylin staining, × 10). (C) Confluent non-necrotizing sarcoid-like granulomas with epithelioid cells and multinucleated giant cells (arrows) are observed (hematoxylin staining, × 20). (D) Vasculitis with lymphoplasmacytic infiltration is also well shown (arrows) (hematoxylin staining, × 40).

We performed a video-assisted thoracoscopic surgery with a lung biopsy of nodules in the left lower lobe. Histopathological evaluation revealed multiple inflammatory nodules with confluent granulomas and large zones of necrosis (Fig. 5). The granulomas were composed of epithelioid cells and multinucleated giant cells. Vasculitis with lymphoplasmacytic infiltration was also well seen. The histopathologic diagnosis was sarcoidosis with NSG pattern.

After the lung biopsy, the attending physician decided medical observation without further treatment because the patient did not reveal any clinical or laboratory abnormality. On follow-up, chest radiography after surgical lung biopsy, pulmonary nodules showed continuous enlargement on a follow-up period of 12 days and otherwise decreased size on a further follow-up after 2 months (figures not shown).

## 3 Discussion

Necrotizing sarcoid granulomatosis, previously considered an extremely rare disease category, is characterized by findings of sarcoid-like granulomas, vasculitis, and a variable degree of necrosis.^[1]^ Histologically, large areas of necrosis involving the lung parenchyma can be seen. They are related to occlusion of pulmonary vessels with granulomatous inflammation. It is unclear whether the sarcoid-like reaction occurred in necrotizing vasculitis or necrosis of the granulomas and vessels in sarcoidosis. In fact, vasculitis accompanying granulomatous lung disease can be seen in various conditions, including GPA, infection such as fungus, tuberculosis or schistosomiasis, and foreign body reaction such as chronic beryllium disease or foreign body pulmonary embolism in drug abusers.^[6,11]^ Recently, it is accepted as a more convincing idea that NSG is a variant of sarcoidosis with extensive infarct-like necrosis.^[9,12,13]^ Therefore, the term “sarcoidosis with NSG pattern” is being used instead of NSG to reflect the clinical entity of nodular sarcoidosis.^[4,7,8]^

Sarcoidosis with NSG pattern can be easily misdiagnosed as pulmonary malignancy or other pulmonary granulomatous diseases due to their similar radiologic findings and nonspecific clinical presentation. Sarcoidosis with NSG pattern has been reported in a wide range of age groups from 8 to 68 years, with a median age of 42 years.^[9]^ It occurs more frequently in women than in men with variable prevalence of extrapulmonary involvement such as eyes, skin, and nerve systems.^[9]^ Karpathiou et al^[9]^ have reviewed clinical, radiologic, and histopathologic findings of NSG and found that the pattern of multiple lung nodules is the most common radiologic presentation, followed by a solitary nodule or mass. Thoracic lymphadenopathy and cavitation of nodules are often observed.^[9]^ Therefore, it is required to distinguish radiologic features of NSG from those of malignancy.

In our case, the initial radiologic findings of the patient were reminiscent of malignant tumors in the sense that they appeared to be enhancing pulmonary nodules with thoracic lymphadenopathy. Clinical features of an asymptomatic patient with incidental radiographic abnormality without specific laboratory finding raised a first impression of malignancy rather than inflammatory diseases. Furthermore, on PET-CT scan, the pulmonary nodules, and lymphadenopathy showed avid FDG uptakes. On retrospective review, microlobulation and low-density areas in the pulmonary nodules of the right middle lobe and the left lower lobe were well correlated with characteristic pathologic findings of aggregates of granulomas and relatively large areas of necrosis. In contrast with frequent central necrosis in metastatic lymph nodes on enhanced chest CT, homogeneously enhancing thoracic lymphadenopathy was found our case. Besides, inflammation can be a plausible explanation considering radiographic change during a month of follow-up period and distribution of pulmonary nodules along bronchovascular bundles.

Various granulomatous lung diseases usually present multiple pulmonary nodules. However, differential radiologic characteristics should be taken into consideration. Nodular sarcoidosis, an uncommon form of sarcoidosis, can show variable sized distinct pulmonary nodules with peripheral predilection or unusually solitary nodule, which simulates metastatic tumor or primary pulmonary neoplasm.^[14]^ Unlike sarcoidosis with NSG pattern, nodular sarcoidosis consists of coalescent compact sarcoid granulomas without significant necrosis or vasculitis on histology. There is still a debate as to whether sarcoidosis with NSG pattern might be a variant or late stage of nodular sarcoidosis. Substantial overlap is seen between nodular sarcoidosis and NSG pattern in terms of clinical symptoms and extrapulmonary manifestations.^[1]^ Patients with NSG pattern are reported to be less likely to have hilar or mediastinal lymphadenopathy with increased serum angiotensin-converting enzyme than those with nodular sarcoidosis.^[1,3,4]^ Vasculitis such as GPA or eosinophilic GPA can also present multiple lung nodules with solid or ground-glass opacity that often show air bronchogram or necrosis with cavitation. Accompanied ground-glass opacity and consolidation are more often seen in GPA than other granulomatous diseases due to alveolar hemorrhage resulting from necrotic infiltration or perfusion alterations by vasculitis of small vessels.^[15,16]^ Extrapulmonary involvement is a common manifestation. It is particularly accompanied by upper respiratory tract disease, glomerulonephritis, or systemic vasculitis. Amyloidosis is also one of the diseases to be distinguished from other granulomatous diseases. Multiple nodular parenchymal amyloid deposits often show central or irregular patterns of calcification with sharp and lobulated margins. Circumferential wall thickening of trachea is a frequent radiologic finding of airway involvement. Less commonly, diffuse involvement of lung parenchyma including capillary vessels and interstitial alveolar septae can be seen.^[17]^

The most commonly used treatment for sarcoidosis with NSG pattern is systemic steroid therapy (daily prednisone 40–60 mg for 4–8 weeks).^[13]^ Unlike other systemic vasculitis or malignancy, good response with systemic corticosteroid treatment is expected. The disease also can show spontaneous regression.^[3]^ This was well demonstrated in our case. Overall prognosis of sarcoidosis with NSG pattern is favorable,^[3]^ which is a notable distinguishing feature in comparison with other differential disease entities. Therefore, minimally invasive diagnostic procedure can be considered for better management of the patient.

## 4 Conclusion

There have been many pathological reports of sarcoidosis with NSG pattern.^[3,9,11,13]^ However, only a few articles on its radiologic characteristics have been reported. In our case report, major radiological findings of sarcoidosis with NSG pattern were discussed with radiologic and pathologic correlation. Distinguishing characteristics from pulmonary malignancies and other granulomatous diseases were also reviewed. Recognizing and understanding the clinical and radiologic presentations of sarcoidosis with NSG pattern can lead to appropriate diagnosis and management. Further large case studies are needed to specify and better define this rare disease entity.

## Author contributions

BDN was the guarantor of the entire study. JHK, BDN, JHH contributed to the conception, design, and acquisition of data, analysis and interpretation of data, and drafting of the paper. DWK contributed to the data collection, analysis, and interpretation of data and drafting of the paper. KUK contributed to the design and acquisition of data, analysis and interpretation of data. YWP contributed to the data collection, analysis, and interpretation of data and drafting of the paper.

**Conceptualization:** Bo Da Nam, Jung Hwa Hwang.

**Supervision:** Jun Hyeok Kim, Bo Da Nam, Jung Hwa Hwang, Dong Won Kim, Ki-Up Kim, Young Woo Park.

**Writing – original draft:** Jun Hyeok Kim, Bo Da Nam, Jung Hwa Hwang.

**Writing – review & editing:** Jung Hwa Hwang.
